# Dual‐Selection Strategy for Generating Knock‐Out Lines of Human Embryonic Stem Cells

**DOI:** 10.1111/jcmm.70259

**Published:** 2025-01-19

**Authors:** Ziyu Zhou, Lingling Tong, Yunbing Chen, Ruoming Wang, Yu Shen, Di Chen

**Affiliations:** ^1^ Center for Reproductive Medicine of the Second Affiliated Hospital, Center for Regeneration and Cell Therapy of Zhejiang University‐University of Edinburgh Institute (ZJU‐UoE Institute), Zhejiang University School of Medicine, Zhejiang University Hangzhou Zhejiang China; ^2^ State Key Laboratory of Biobased Transportation Fuel Technology Haining Zhejiang China; ^3^ Dr. Li Dak sum & yip Yio Chin Center for Stem Cell and Regenerative Medicine, Zhejiang University Hangzhou Zhejiang China

**Keywords:** CRISPR/Cas9, dual selection, human embryonic stem cells, Knock‐out

Human pluripotent stem cells (hPSCs), including human embryonic stem cells (hESCs) and induced pluripotent stem cells (hiPSCs), possess the capacity to differentiate into all the cell types in the adults, forming the basis for generating functional cells in vitro for regenerative medicine [1]. The purity and functionality of the hPSC‐derived cells are the two key factors for clinical applications, which are largely determined by the differentiation protocols and conditions. The generation of knock‐out lines of hPSCs is critical for dissecting the functions of the genes‐of‐interest and investigating the molecular mechanisms involved, essential for optimising differentiation protocols towards different lineages [2, 3]. The development of the CRISPR/Cas9 technique has greatly improved the efficiency of gene targeting, bursting the functional analysis of genes‐of‐interest in hPSCs [4, 5]. Moreover, the development of stem cells‐ and hPSCs‐based organoid platforms further necessitated the genomic engineering for generating knock‐out mutants and knock‐in reporters [6, 7]. However, the efficiency of gene editing in hPSCs is low and the whole process is tedious [4, 8, 9].

To reduce the workload and increase the efficiency of gene targeting for hPSCs, we designed a dual‐selection strategy that incorporates antibiotic selection and fluorescent enrichment. Two donor vectors for homologous recombination were constructed, one with *GFP‐2A‐drug resistant gene* (*DRG*) and the other with *RFP‐2A‐DRG*. A pair of guide RNAs (gRNAs) was designed to delete the whole region of the gene‐of‐interest. Two donor vectors served as templates for homologous recombination–based DNA repair. In a few cells, the genomic region of the gene‐of‐interest could be replaced with one allele of *GFP‐2A‐DRG* and the other allele of *RFP‐2A‐DRG*, forming the basis for antibiotic selection and fluorescent selection for a successful knock‐out cell population. Furthermore, the cassettes *of GFP‐2A‐DRG* and *RFP‐2A‐DRG* are flanked by loxP. Both could be removed after the expression of Cre (Figure 1A). Thus, mutant hPSC lines could be easily selected by one round of antibiotic selection and another round of fluorescent enrichment.

**FIGURE 1:**
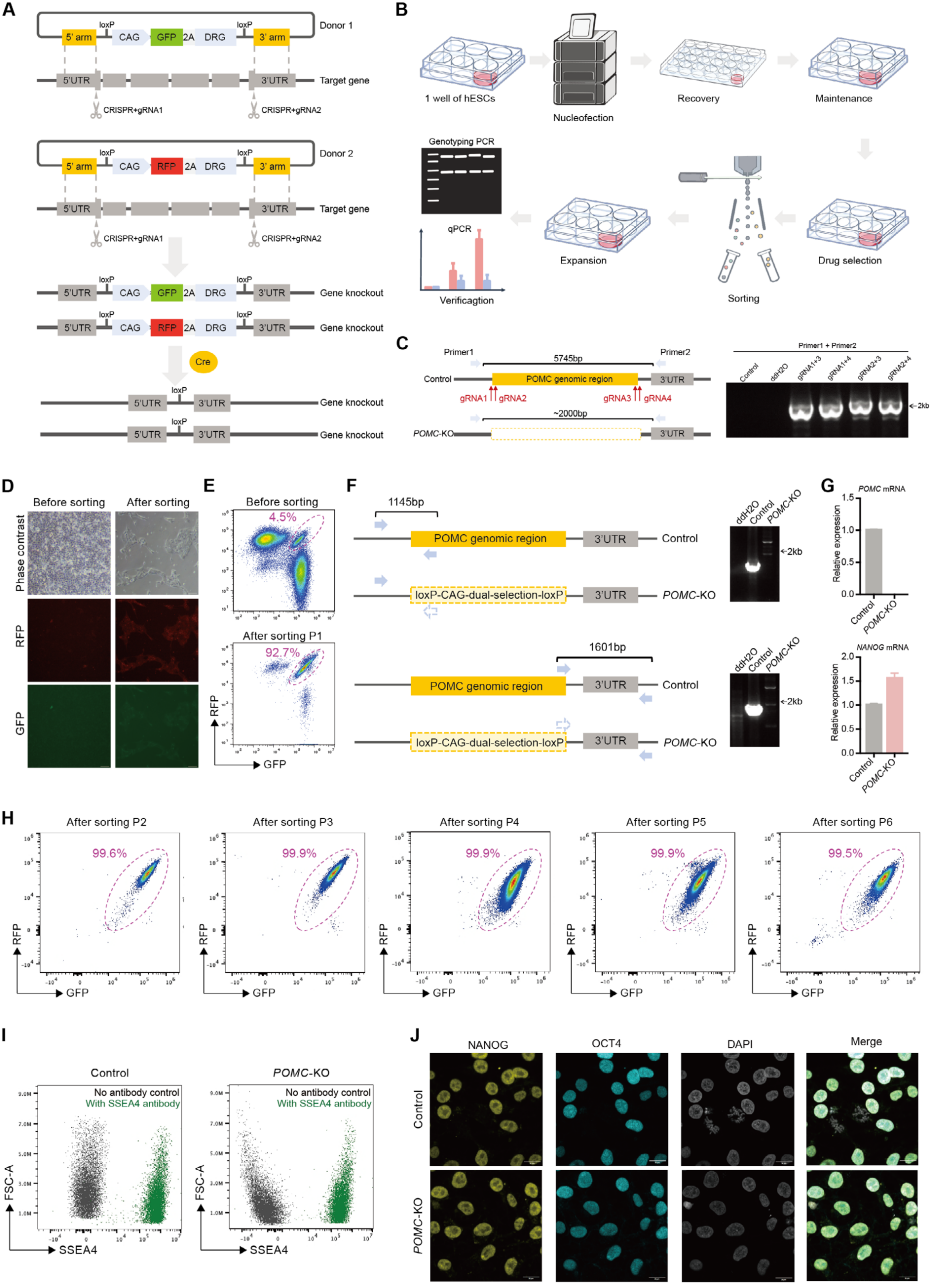
| Dual‐selection strategy for generating knock‐out lines of hESCs. (A) Schematic illustration of the principle of dual‐selection strategy. A pair of gRNAs is designed to delete the whole genomic region of gene of interest. Two donor vectors (one with GFP and the other with RFP) are designed with about 1 kilobase (kb) of 5′ homologous arm (5’arm) and about 1 kb of 3′ homologous arm (3’arm) for homologous recombination to be knocked into the locus of gene‐of‐interest. The *CAG‐GFP‐2A‐DRG* (drug‐resistant gene) and *CAG‐RFP‐2A‐DRG* are flanked with loxP and could be removed by the expression of Cre recombinase. (B) The main experimental steps for generating knock‐out lines of human embryonic stem cells using the dual‐selection method. (C) Genotyping PCR for screening the best combination of gRNAs for cutting the *POMC* gene. (D) Phase contrast and fluorescent images of hESCs before and after sorting. Scale bar: 100 μm. (E) Flow cytometry plots showing the GFP and RFP double‐positive cells before and after sorting. KO: Knock‐out. (F) Genotyping PCR for validating the successful knock‐out of the *POMC* gene. (G) Quantitative PCR (qPCR) confirming the depletion of the *POMC* gene. (H) Flow cytometry analysis of GFP and RFP double‐positive *POMC*‐KO cells from passages 2–6 after sorting. (I) Flow cytometry showing the expression of SSEA4 in control and *POMC*‐KO cells. (J) Immunofluorescence showing the expression of NANOG, OCT4 and DAPI in control and *POMC*‐KO cells. Scale bar: 20 μm.

One of the most time‐consuming steps for generating knock‐out lines in hPSCs is the screening of homozygous mutants. According to our experience, the efficiency for generating homozygous mutants of gene‐of‐interest in hESCs was usually less than 5% [10, 11]. Therefore, more than 100 single colonies were required to be individually picked, expanded, passaged and genotyped by at least two rounds of polymerase chain reaction (PCR). With the dual‐selection strategy, 800,000 single cells were nucleofected with two donor vectors and a pair of gRNAs deleting the genomic region of gene‐of‐interest. The nucleofected cells were recovered and expanded for drug selection. The expanded drug‐resistant cells were enriched as GFP and RFP double‐positive cells by fluorescence‐activated cell sorting (FACS). These drug‐resistant and fluorescence‐positive cells were expanded and verified by genotyping PCR and quantitative PCR (qPCR) or other methods such as western blot or immunofluorescence if suitable antibodies are available (Figure 1B). Therefore, no single colony‐manipulation‐related experiment is required, dramatically reducing the workload for screening out the homozygous knock‐out hPSC lines.

To test this dual‐selection method, we chose the *Pro‐opiomelanocortin* (*POMC*) gene. *POMC* is expressed in specific neurons critical for metabolic homeostasis and is related to diseases such as obesity [12, 13]. *POMC* knock‐out hESC lines may serve as disease models for functional analysis of *POMC* and pathogenic investigation of *POMC*‐related diseases. First, we designed two pairs of gRNAs to screen for the combination of gRNAs that would cut the genomic region of *POMC* with CRISPR/Cas9 efficiently. The efficiency of the different combinations was determined by transfecting all four combinations of gRNAs into HEK293T cells, followed by genotyping PCR after 2 days (Figure 1C). The genotyping primers were designed to amplify a ~ 2 kilobase (kb) band after cutting with Cas9. We found that all four combinations resulted in the ~2 kb bands (Figure 1C), suggesting that all the combinations function to cut the genomic region of *POMC* efficiently. We then chose the combination of gRNA1 and gRNA2 for subsequent experiments in hESCs.

First, all the components including CRISPR/Cas9, gRNA1‐2, and the two donor constructs were nucleofected into hESCs. Given the low efficiency of CRISPR/Cas9 cutting and donor replacement, there were very few cells that showed GFP or RFP signal 1 day after nucleofection (Figure 1D). After antibiotic selection for a few days, the cells underwent FACS, resulting in about 4.5% of the cells being double positive for GFP and RFP (Figure 1E). Importantly, almost all cells were double positive for GFP and RFP after sorting (Figure 1D,E), suggesting that this dual‐selection method could enrich the hESCs with both alleles replaced by the donor constructs. Next, we verified the knock‐out of *POMC* (*POMC*‐KO) gene by genotyping PCR. Two pairs of primers were designed with one primer from each pair located outside the deleted region, while the other primer from each pair was located inside the deleted region (Figure 1F). Therefore, no PCR band was detected when both alleles of *POMC* were deleted (Figure 1F). To further verify the deletion of *POMC* genes, we conducted qPCR using primers targeting the coding region of *POMC* and discovered that indeed both alleles of *POMC* were deleted (Figure 1G). Furthermore, we conducted a series of flow cytometry analyses for five successive passages, monitoring the stability of the double‐positive cell population. The results indicated that over 99% of the cells remained double‐positive throughout these passages (Figure 1H), underscoring the stability and reliability of our strategy. Lastly, we assessed whether this dual‐selection strategy affected the pluripotency of the hESCs. We first examined the expression of the pluripotency marker SSEA4. The *POMC*‐KO line retained comparable levels of SSEA4 expression to the control (Figure 1I). Additionally, we performed immunofluorescence for key pluripotency markers and discovered that the *POMC*‐KO cells were positive for NANOG and OCT4 (Figure 1J), confirming the pluripotent state of the *POMC*‐KO hESCs. Collectively, we demonstrated that we have successfully generated hESCs with homozygous deletion of *POMC* using the dual‐selection strategy.

To further validate the robustness of our dual‐selection strategy, we applied it to generate homozygous deletion of *methyltransferase‐like protein 14* (*METTL14)* in hESCs. Similar to the strategy for *POMC* (Figure 1A,B), we designed gRNAs targeting *METTL14* and corresponding donors. After antibiotic selection, we performed FACS and identified approximately 19.9% of cells as double positive for GFP and RFP (Figure 2A,B). The successful knock‐out of *METTL14* was confirmed by genotyping PCR (Figure 2C), qPCR (Figure 2D), and immunofluorescence (Figure 2E,F), confirming the depletion of *METTL14*. Next, we compared the *METTL14* knock‐out (*METTL14*‐KO) hESCs with and without the loxP‐flanked *GFP‐2A‐DRG* and *RFP‐2A‐DRG* cassettes (Figure 2E,F). To achieve this, *METTL14*‐KO hESCs with *GFP‐2A‐DRG* and *RFP‐2A‐DRG* were nucleofected with plasmid DNA expressing Cre to establish *METTL14*‐KO hESC sublines without GFP or RFP after colony purification (Figure 2E,F). To determine whether the selection markers (*GFP‐2A‐DRG* and *RFP‐2A‐DRG*) may affect pluripotency or proliferation of hESCs, we compared the following three groups: *METTL14*‐KO with GFP & RFP, *METTL14*‐KO without GFP & RFP, and control hESCs. All these three groups of cells were positive for pluripotency marker SSEA4 (Figure 2G) and exhibited comparative percentages of EdU‐positive cells (Figure 2H,I), indicating that the insertion of *GFP‐2A‐DRG* or *RFP‐2A‐DRG* cassettes has no effect on the pluripotency or proliferation of the knock‐out cells. Taken together, we have successfully generated *METTL14*‐KO hESCs with and without selection cassettes using dual‐selection strategy.

**FIGURE 2:**
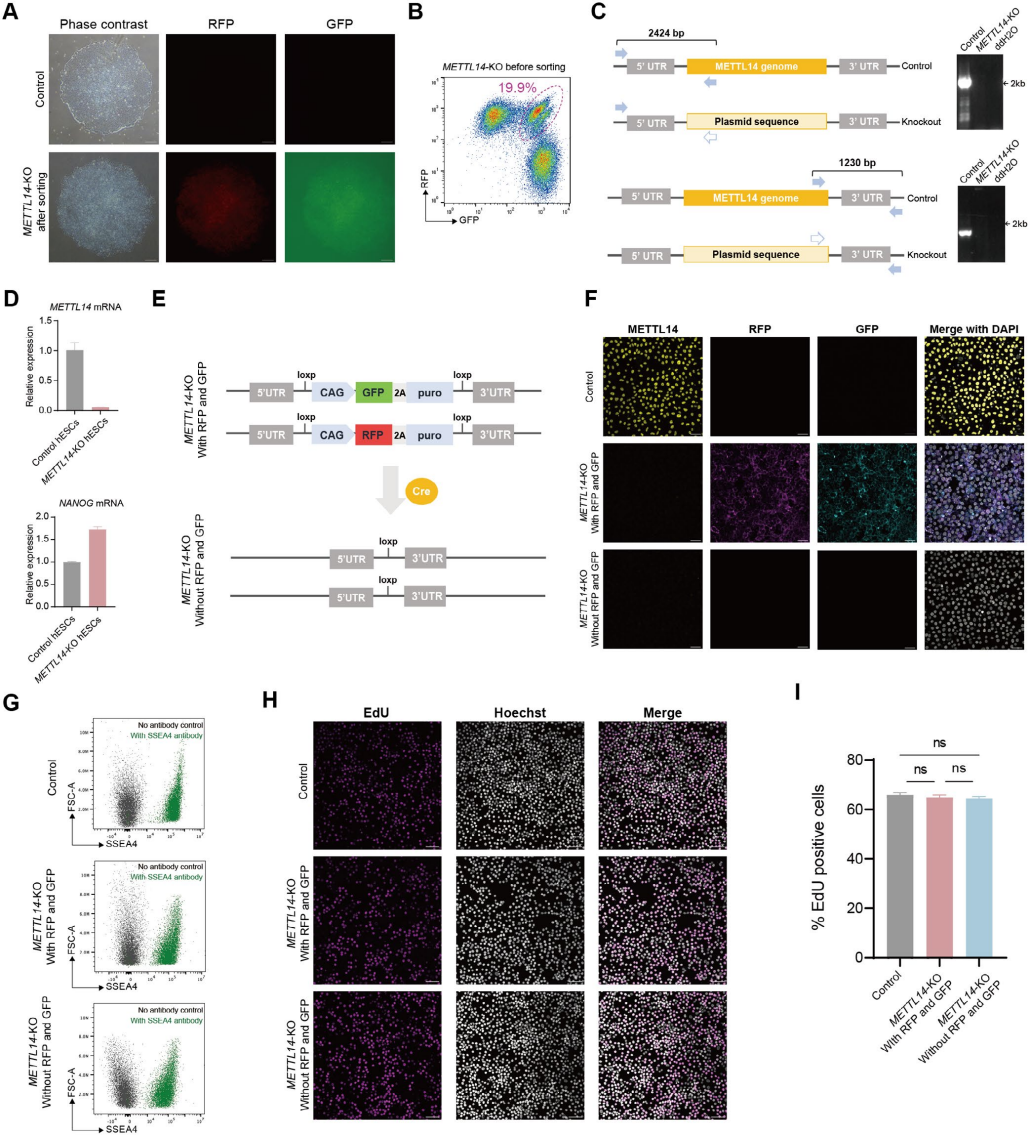
| Generation of *METTL14* knock‐out hESC lines. (A) Phase contrast and fluorescent images of hESCs before and after sorting. Scale bar: 100 μm. (B) Flow cytometry plots showing the GFP and RFP double‐positive cells before and after sorting. (C) Genotyping PCR for validating the successful knock‐out of the *METTL14* gene. (D) qPCR experiments confirming the depletion of the *METTL14* gene. (E) Schematic illustration of Cre‐loxP system for removing fluorescent and antibiotic selection markers from *METTL14*‐KO cell lines. (F) Immunofluorescence showing the expression of METTL14, GFP and RFP in control, *METTL14*‐KO with GFP & RFP and *METTL14*‐KO without GFP & RFP hESCs. DAPI is counterstained for nuclei. Scale bar: 50 μm. (G) Flow cytometry analysis of *SSEA4* expression in control, *METTL14*‐KO with GFP & RFP and *METTL14*‐KO without GFP & RFP hESCs. (H‐I) EdU incorporation assay and quantification for control, *METTL14*‐KO with GFP & RFP, and *METTL14*‐KO without GFP & RFP hESCs. Scale bar: 100 μm.

Next, we used these fluorescence‐labelled knock‐out hESCs for organoid induction. We applied the GFP and RFP double‐positive *POMC*‐KO hESCs for organoid differentiation towards endodermal, mesodermal and ectodermal cells [14, 15]. After 6–8 days of organoid culture, we observed clear 3D structure and both GFP and RFP signals in endodermal, mesodermal and ectodermal spheres (Figure 3A–C). Importantly, *POMC* is upregulated towards endoderm differentiation, while downregulated towards mesodermal or ectodermal differentiation (Figure 3D). To verify the germ layer differentiation, we applied qPCRs for germ layer‐specific markers. The endodermal organoids expressed endoderm markers such as *SOX17*, *FOXA2,* and *GATA6* (Figure 3E); the mesodermal organoids expressed mesoderm markers such as *TBXT*, *MIXl1,* and *SP5* (Figure 3F); and the ectodermal organoids expressed ectoderm markers including *PAX6*, *SOX1,* and *HES5* (Figure 3F), indicating the successful germ layer differentiation. Interestingly, endodermal and mesodermal markers were upregulated in *POMC*‐KO endoderm and mesoderm organoids respectively (Figure 3E,F). However, ectodermal markers were downregulated in ectoderm organoids (Figure 3G). These observations indicate that *POMC* is probably involved in germ layer differentiation, especially for ectoderm differentiation, consistent with the discovery that *POMC* is involved in neural development [12, 13]. Therefore, organoids generated from GFP and RFP double‐positive *POMC* knock‐out hESCs could be applied for functional dissections and disease modelling.

**FIGURE 3:**
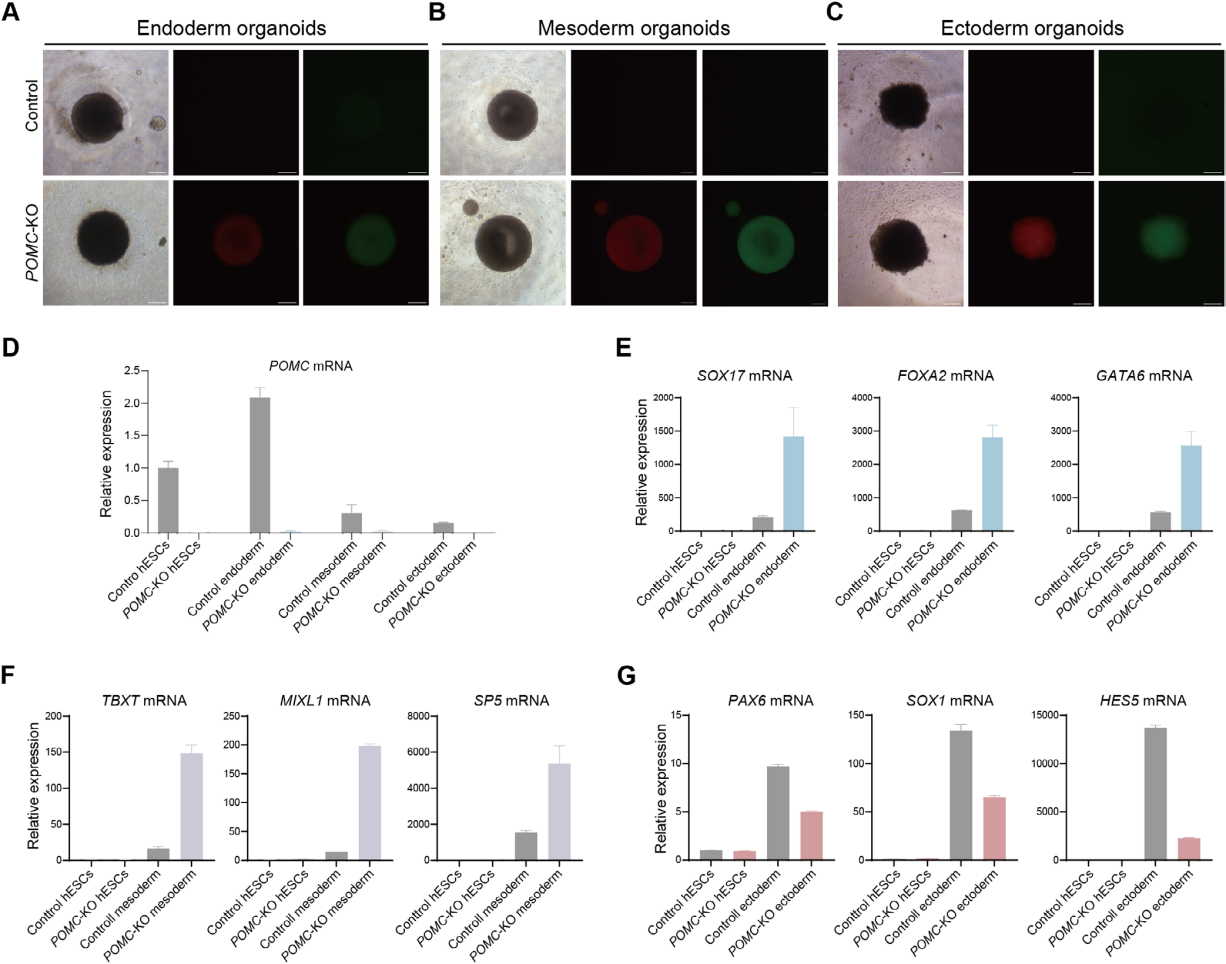
| Induction of organoids from *POMC*‐KO hESCs. (A‐C) Organoid formation towards endoderm, mesoderm and ectoderm differentiation using control and *POMC*‐KO hESCs. 20,000–40,000 single hESCs were plated on the low‐attached 96‐well plate for generating organoids for differentiation towards endoderm, mesoderm and ectoderm. Scale bar: 100 μm. (D) qPCR determination of the expression of *POMC* in control and *POMC*‐KO hESCs, endoderm organoids, mesoderm organoids and ectoderm organoids. (E) qPCR determination of the expression of *SOX17*, *FOXA2* and *GATA6* in control and *POMC*‐KO hESCs and endoderm organoids. (F) qPCR determination of the expression of *TBXT*, *MIXL1* and *SP5* in control and *POMC*‐KO hESCs and mesoderm organoids. (G) qPCR determination of the expression of *PAX6*, *SOX1* and *HES5* in control and *POMC*‐KO hESCs and ectoderm organoids.

Overall, this dual‐selection strategy highly reduced the workload by avoiding the single colony picking and genotyping for each individual subclonal line. Besides, this dual‐selection strategy reduced the rate of false‐positive cells after two rounds of selection. Furthermore, it could be applied to generate double, triple or even more genes‐of‐interested deleted in one cell line round by round. One important advantage of this dual selection is that the knock‐out hESCs are double positive for GFP and RFP. GFP and RFP could serve as markers to label the knock‐out cells for stem cell differentiation, live imaging, and xeno‐transplantation. Given that the dual‐selection cassettes are flanked by loxP sites, these cassettes could be removed by overexpression of Cre. In summary, we have designed a dual‐selection strategy to generate genome‐edited hESC lines with highly reduced workload and improved efficiency, removable fluorescence for cell tracing, and potentials for upgrading to achieve endogenous tagging for reporters and degrons.

## Funding Information:

This work was supported by the National Natural Science Foundation of China awarded to D.C. (Grant No. 32270835), Zhejiang Natural Science Foundation awarded to D.C. (Grant No. Z22C129553) and Dr. Li Dak Sum & Yip Yio Chin Development Fund for Regenerative Medicine, Zhejiang University, awarded to DC.

## Author Contributions

**Ziyu Zhou** took part in data curation (lead), formal analysis (equal), investigation (equal), validation (equal) and writing original draft (equal). **Lingling Tong** participated in data curation (equal), formal analysis (equal), investigation (equal), validation (equal) and writing original draft (equal). **Yunbing Chen** contributed to data curation (equal), formal analysis (equal), investigation (equal), validation (equal) and writing original draft (equal). **Ruoming Wang** took part in investigation (equal) and validation (equal). **Yu Shen** participated in investigation (equal). **Di Chen** contributed to conceptualisation (lead), data curation (equal), funding acquisition (lead), investigation (supporting), validation (supporting), project administration (equal), resources (lead), supervision (lead) and writing original draft (equal).

## Conflicts of Interest

The authors confirm that there are no conflicts of interest.

## Supporting Information

Additional supporting information can be found online in the Supporting Information section at the end of this article.

## Data Availability

The raw data supporting the conclusions of this article will be made available by the authors, without undue reservation.
